# EGFR-L858R mutant enhances lung adenocarcinoma cell invasive ability and promotes malignant pleural effusion formation through activation of the CXCL12-CXCR4 pathway

**DOI:** 10.1038/srep13574

**Published:** 2015-09-04

**Authors:** Meng-Feng Tsai, Tzu-Hua Chang, Shang-Gin Wu, Hsiao-Yin Yang, Yi-Chiung Hsu, Pan-Chyr Yang, Jin-Yuan Shih

**Affiliations:** 1 Department of Molecular Biotechnology, College of Biotechnology and Bioresources, Dayeh University, Changhua 51591, Taiwan; 2 Department of Internal Medicine, National Taiwan University Hospital, and College of Medicine, National Taiwan University, Taipei 10002, Taiwan; 3 Department of Internal Medicine, National Taiwan University Hospital, Yun-Lin Branch, Yunlin 64041, Taiwan; 4 Graduate Institute of Clinical Medicine, College of Medicine, National Taiwan University, Taipei 10002, Taiwan; 5 Institute of Statistical Science, Academia Sinica, Taipei 11529, Taiwan

## Abstract

Malignant pleural effusion (MPE) is a common clinical problem in non-small cell lung carcinoma (NSCLC) patients; however, the underlying mechanisms are still largely unknown. Recent studies indicate that the frequency of the L858R mutant form of the epidermal growth factor receptor (EGFR-L858R) is higher in lung adenocarcinoma with MPE than in surgically resected specimens, suggesting that lung adenocarcinoma cells harboring this mutation tend to invade the adjacent pleural cavity. The purpose of this study was to clarify the relationship between the EGFR-L858R mutation and cancer cell invasion ability and to investigate the molecular mechanisms involved in the formation of MPE. We found that expression of EGFR-L858R in lung cancer cells resulted in up-regulation of the CXCR4 in association with increased cancer cell invasive ability and MPE formation. Ectopic expression of EGFR-L858R in lung cancer cells acted through activation of ERK signaling pathways to induce the expression of CXCR4. We also indicated that Inhibition of CXCR4 with small interfering RNA, neutralizing antibody, or receptor antagonist significantly suppressed the EGFR-L858R–dependent cell invasion. These results suggest that targeting the production of CXCR4 and blocking the CXCL12-CXCR4 pathway might be effective strategies for treating NSCLCs harboring a specific type of EGFR mutation.

Lung cancer is the most common cause of cancer death in the world, and patients with this disease have a 5-year survival rate less than 15%^1^,^2^. Non-small cell lung carcinoma (NSCLC), the predominant histological type of lung cancer, accounts for nearly 85% of lung cancer cases^1^. End-stage NSCLC is associated with distant metastasis and the formation of malignant pleural effusion (MPE), with the latter being a significant source of cancer-related morbidity. Approximately 15% of lung cancer patients have MPE at the time of initial diagnosis, and 50% develop it later in the course of their disease^3^,^4^. The presence of pleural effusion in patients with NSCLC usually indicates advanced disease and portends a grave prognosis^5^.

The production of MPE reflects cancer cell invasion into the pleura and accumulation of fluid within the pleural space owing to increased vascular permeability and leakage. MPE is diagnosed by the identification of malignant cells in pleural fluid or upon pleural biopsy^6^,^7^. Although almost all types of cancers can cause MPE, more than 75% of MPEs are attributable to metastases originating from lymphomas or tumors in the lung, breast, or ovary^6^,^8^. Notably, the shortest survival time is observed among lung cancer patients with MPE^8^,^9^,^10^. Patients with MPE have a poor prognosis and are difficult to treat effectively^9^,^11^. The standard treatment of MPE is evacuation of the pleural fluid, followed by pleurodesis with instillation of antibiotics, antiseptics, or antineoplastics^12^,^13^. Most previous management strategies focused on symptom control and did not improve patient survival. However, in the modern era of targeted therapy, clinicians have the option of treating lung adenocarcinoma patients harboring EGFR mutations with EGFR tyrosine kinase inhibitors (TKIs), which have improved the survival of such patients. EGFR mutations can be detected in cancer cells of MPEs, and are useful for predicting the response to the TKIs, as shown in previous studies by us and others^14^,^15^,^16^. Patients with lung adenocarcinoma-associated MPE have an increased frequency of EGFR mutations^15^,^17^,^18^. Patients with stage IV lung adenocarcinoma with MPE at initial diagnosis have a shorter overall survival and higher rate of EGFR mutations, especially L858R, than patients who develop MPE following disease progression^14^. From these observations, it has been postulated that mutation of the EGFR is an early event in the pathogenesis of lung adenocarcinoma. In particular, the EGFR-L858R mutation may play a role in the development of MPE in lung adenocarcinoma patients.

Although the pathogenesis of MPE is not fully understood, it is generally thought to involve tumor metastasis, angiogenesis, lymphangiogenesis, and tumor-associated inflammation^19^,^20^,^21^,^22^. Vascular endothelial growth factor (VEGF), a potent mediator of endothelial permeability, has been implicated as a critical cytokine in MPE pathogenesis^23^. In addition to VEGF, other cytokines are also detected in MPEs, including the interleukins IL21 and IL17, and the C-C and C-X-C motif chemokine ligands CCL2 and CXCL12 (also known as SDF-1α), respectively^11^,^24^,^25^,^26^. MPE is a common clinical problem for patients with lung adenocarcinoma, but its causes and underlying mechanisms are still largely unknown. A better understanding of the molecular mechanisms that regulate the pathogenesis of the MPE process could lead to the design of novel, effective therapies for these patients.

The purpose of this study was to clarify the relationship between the EGFR-L858R mutation and cancer cell invasion ability and to determine the molecular mechanisms involved in the formation of MPE. Our findings demonstrate that lung adenocarcinomas harboring the EGFR-L858R mutation exhibit increased cancer cell invasive ability and MPE formation through activation of the CXCL12-CXCR4 axis.

## Results

### Expression of EGFR-L858R in lung cancer cells

**Figure 1:**
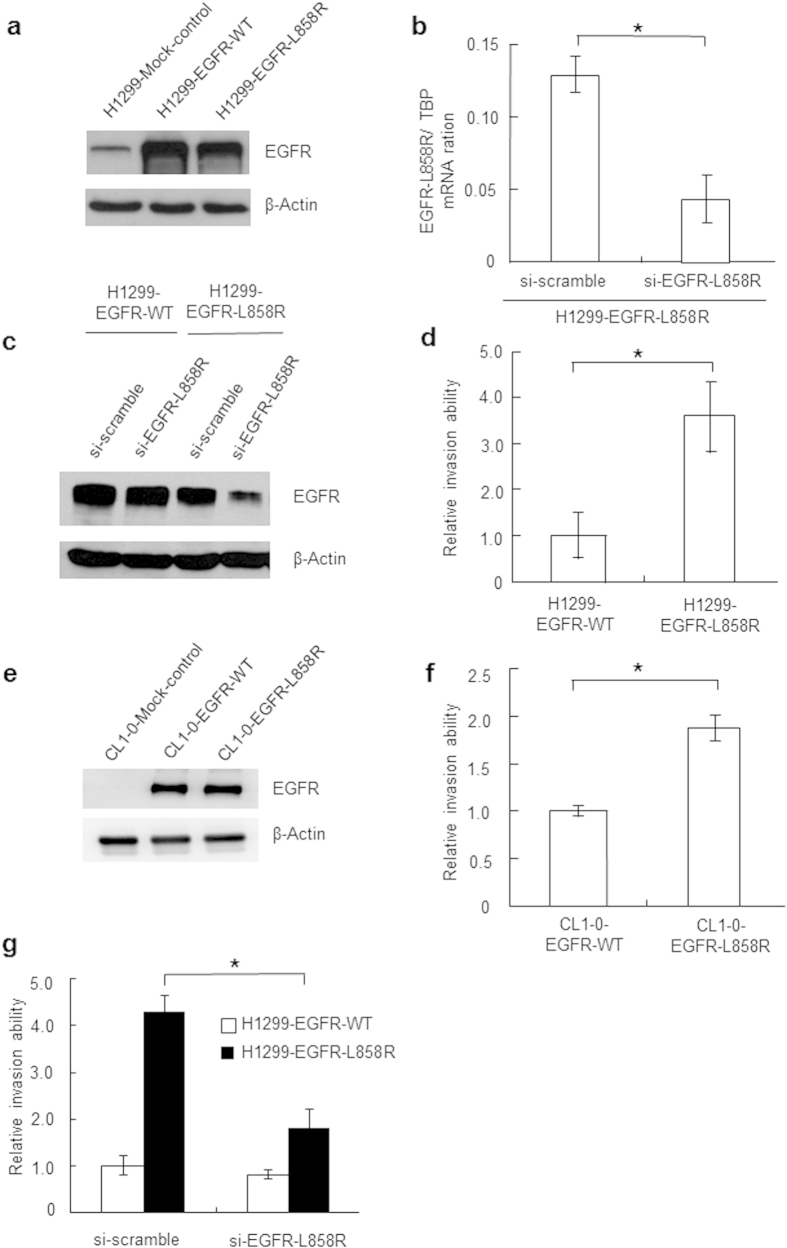
EGFR-L858R expression and lung cancer cell invasion ability. (**a**) Over-expression of EGFR and EGFR-L858R in lung adenocarcinoma H1299 cells was evaluated by Western blotting. H1299-EGFR-WT and H1299-EGFR-L858R cells were stably transfected with EGFR-WT and EGFR-L858R, respectively; H1299-mock was a mock-transfected cell line used as a control. EGFR-L858R expression in H1299-EGFR-L858R cells was knocked down using an EGFR-L858R–specific siRNA. The full size blots were shown in the Supplementary Figure S2 and band of interest is indicated with an arrow. (**b**) EGFR-L858R expression was quantified by real-time RT-PCR (*P < 0.001). (**c**) Using an EGFR-L858R–specific siRNA, EGFR-L858R expression was knocked down in H1299-EGFR-L858R cells. EGFR protein was evaluated by Western blotting. The full size blots were shown in the Supplementary Figure S2 and band of interest is indicated with an arrow. (d) The invasiveness of cells constitutively expressing EGFR-WT or EGFR-L858R in H1299 cells was evaluated using a modified Boyden chamber assay (*P < 0.01). (**e**) CL1-0 cells were transiently transfected with pCDNA4-EGFR-WT and pCDNA4-EGFR-L858R plasmids by using Lipofectamine method. The expression of EGFR-WT and EGFR-L858R in lung adenocarcinoma CL1-0 cells was evaluated by Western blotting. The full size blots were shown in the Supplementary Figure S2 and band of interest is indicated with an arrow. (**f**) The invasiveness of cells expressing EGFR-WT or EGFR-L858R in CL1-0 transfectants was evaluated using a modified Boyden chamber assay (*P < 0.01). (**g**) The invasion ability of H1299-EGFR-WT and H1299-EGFR-L858R cells was determined using a modified Boyden chamber after siRNA-mediated knockdown of EGFR-L858R (*P < 0.01). si-Scramble, cells transfected with scrambled siRNA control; si-EGFR-L858R, cells transfected with EGFR-L858R–specific siRNA. Data are presented as means ± SD of three independent experiments. *P*-values (two-sided) were determined using Student’s t-test.

To assess the role of the EGFR-L858R mutation in cancer cell invasion ability and involvement in the formation of MPE, we used two H1299-derived NSCLC cell lines—H1299-EGFR-WT, overexpressing wild-type EGFR, and H1299-EGFR-L858R, overexpressing EGFR-L858R—as a model system. H1299 cells were selected as permanently transfected cells for wild-type and mutated EGFRs because they express wild-type EGFR and respond well to EGF treatment. Under regular culture condition (10% fetal bovine serum, FBS), all EGFR mutants displayed a higher level of Tyr phosphorylation than did wild-type EGFR in H1299 cells^27^. A Western blot analysis showed that the expression levels of EGFR-WT and EGFR-L858R in the respective cell lines were similar (Fig. 1a). In this study, we also applied the small interfering RNA (siRNA) approach to abolish the expression of EGFR-L858R. EGFR-L858R specific siRNA has been reported previously and designed to target the nucleotide sequence 5′-CACAGATTTTGGGCGGGCCAA-3′. This specific siRNA only targeting the EGFR-L858R missense transcript, but not the EGFR-WT and EGFR-Del-E745-A750^28^. SiRNA specifically targeting EGFR-L858R knocked down EGFR-L858R transcripts in H1299-EGFR-L858R cells by more than 55% and also severely reduced EGFR-L858R protein expression (Fig. 1b,c). Here, we also indicated that the EGFR-L858R–specific siRNAs only minimal or not affect the protein levels of EGFR-WT in H1299-EGFR-WT cells (Fig. 1c).

### EGFR-L858R mutation and lung cancer cell invasion ability

The cell proliferation rate were detected by MTT assay. There was no significant difference in the cell proliferation rates between H1299-EGFR-WT and H1299-EGFR-L858R cells (Supplementary Figure S1). We used a modified Boyden chamber assay to investigate whether the EGFR-L858R mutation affected the invasive behavior of lung cancer cells. H1299-EGFR-L858R cells incubated for 18 hours exhibited significantly greater invasive activity compared with control H1299-EGFR-WT cells (P < 0.01, Fig. 1d). To validate these findings, we performed these experiments on another lung cancer cell line CL1-0 using transiently transfection methods. The expressions of EGFR-WT and EGFR-L858R in CL1-0 transfectants were detected by Western blot analysis (Fig. 1e). The results of cell invasion assay also revealed that transiently transfected EGFR-L858R mutant in CL1-0 lung cancer cells can promoted the lung cancer invasion ability (P < 0.01, Fig. 1f). In addition, transfection with EGFR-L858R-specific siRNA suppressed EGFR-L858R protein expression and inhibited cell invasion ability in H1299-EGFR-L858R cells (Fig. 1c,g). These observations indicate that the EGFR-L858R mutant promotes lung cancer invasion ability.

### EGFR-L858R mutation and formation of MPE

**Figure 2:**
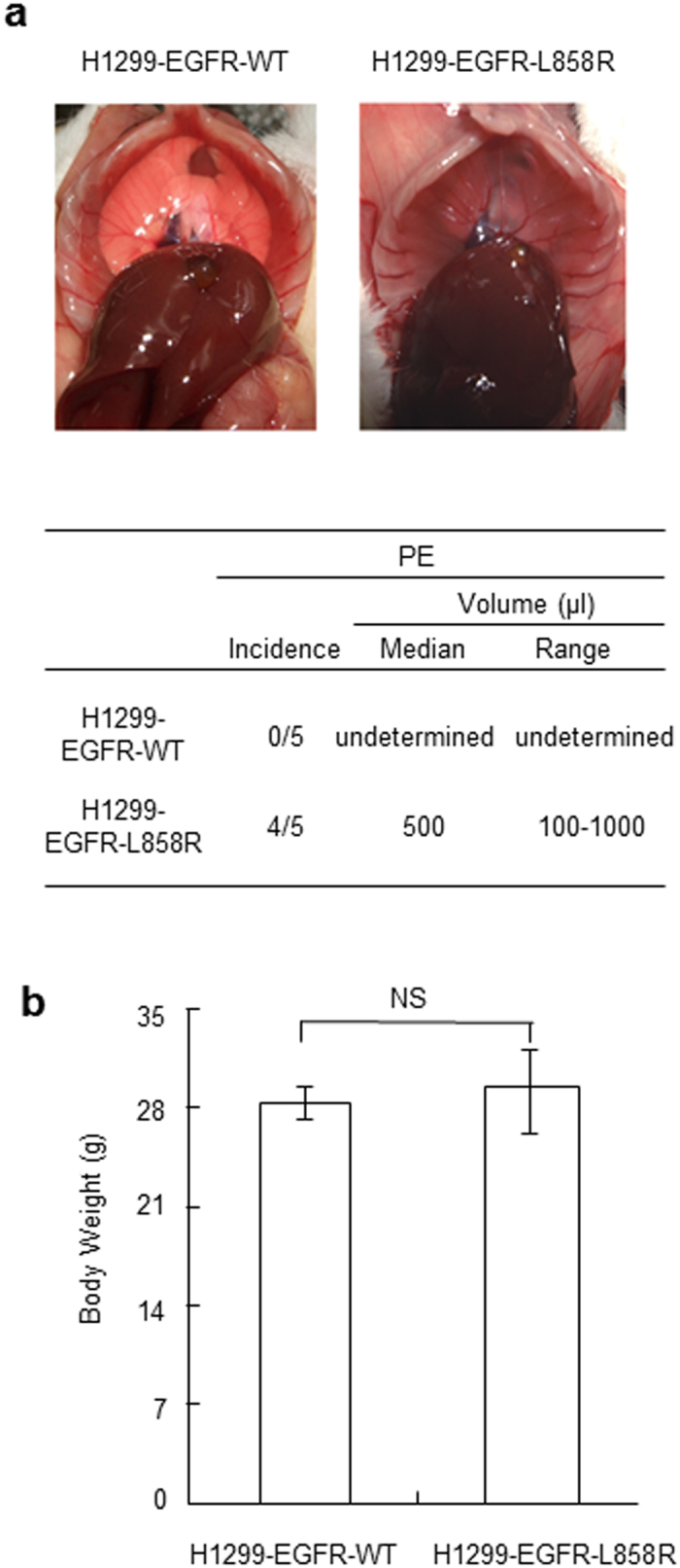
The EGFR-L858R mutant induces production of MPE in the NOD/SCID mouse model. (A) Mice (n = 5/group) received an intrapleural injection of H1299-EGFR-WT or H1299-EGFR-L858R cells, and were sacrificed on day 14. MPE formation was markedly increased in H1299-EGFR-L858R cells compared with H1299-EGFR-WT cells 14 days after injection. (**a**) A right panel of trans-diaphragmatic view of NOD/SCID mouse showing MPE accumulation. (**b**) Body weight measurements. NS, no significant difference.

To investigate whether the EGFR-L858R mutation plays an important role in the formation of MPE, the animal model of intrapleural injections were performed^29^,^30^. Intrapleurally injected H1299-EGFR-L858R or control H1299-EGFR-WT cells into NOD/SCID mice. As shown in Fig. 2a, H1299-EGFR-L858R cells markedly increased the formation of MPE at day 14 after intrapleural injection compared with that produced by control H1299-EGFR-WT cells. Trans-diaphragmatic view of NOD/SCID mouse 14 days after intrapleural injection of H1299-EGFR-WT cells, the mouse lungs are visible due to lack of fluid (left panel). The final body weights in these two groups were similar (Fig. 2b). When 1.5 × 10^5^ H1299-EGFR-L858R or control H1299-EGFR-WT cells were injected, 100% of the animal yielded pleural tumors. The size and the number of the pleura tumor has no significantly difference between these two groups. All of the pleural tumors obtained from this study were less than 2 mm. There are no lung nodules and distant metastasis were observed. However, control H1299-EGFR-WT cells failed to promote the development of MPE in 100% of mice tested (5/5) at 14 days after inoculation, whereas H1299-EGFR-L858R cells promoted the development of pleural effusion in 80% (4/5) of mice, with volumes ranging from 100 to 1000 μl. Collectively, these results indicate that the lung cancer cells harbored the EGFR-L858R mutant promotes the formation of MPE *in vivo*.

### EGFR-L858R mutation and expression of chemokine receptors

Tumor cell migration and metastasis share many similarities with leukocyte trafficking, which is critically regulated by chemokines and their receptors^31^. Accumulating recent evidence points to the vital effects of several chemokines on the proliferative and invasive properties of cancer cells and the pathogenesis of MPE^25^,^26^,^31^,^32^. To further investigate the molecular mechanisms involved in the formation of MPE in lung adenocarcinomas harboring the EGFR-L858R mutation, we first determined whether chemokine receptors were differentially expressed between H1299-EGFR-L858R and H1299-EGFR-WT cells by performing the gene expression profile GSE11729^33^ from the Gene Expression Omnibus (GEO) public database. In the absence of EGF stimulation, mRNA levels of three chemokine receptors—CXCR4, CXCR5 and CXCR6—were up-regulated to varying degrees in the H1299-EGFR-L858R cells compared to control H1299-EGFR-WT cells. After stimulation with EGF, CXCR4 and CX3CR1 mRNA levels were increased at least 2-fold in H1299-EGFR-L858R cells compared with H1299-EGFR-WT cells (Table 1). CXCR4 mRNA levels were increased 2.6-fold, while CXCR6 mRNA levels were increased less than 1.5-fold in H1299-EGFR-L858R cells after EGF stimulated. Since tyrosine phosphorylation on the EGFR mutants could be further increased by EGF stimulation, suggesting that the mutant EGFRs exhibit both ligand-independent and ligand-dependent activation^34^,^35^. In this analysis only CXCR4 mRNA levels were simultaneously up-regulated in the H1299-EGFR-L858R cells with or without EGF stimulation. Therefore, we focus on CXCR4 for further study.

### The EGFR-L858R mutant increases CXCR4 expression

**Figure 3:**
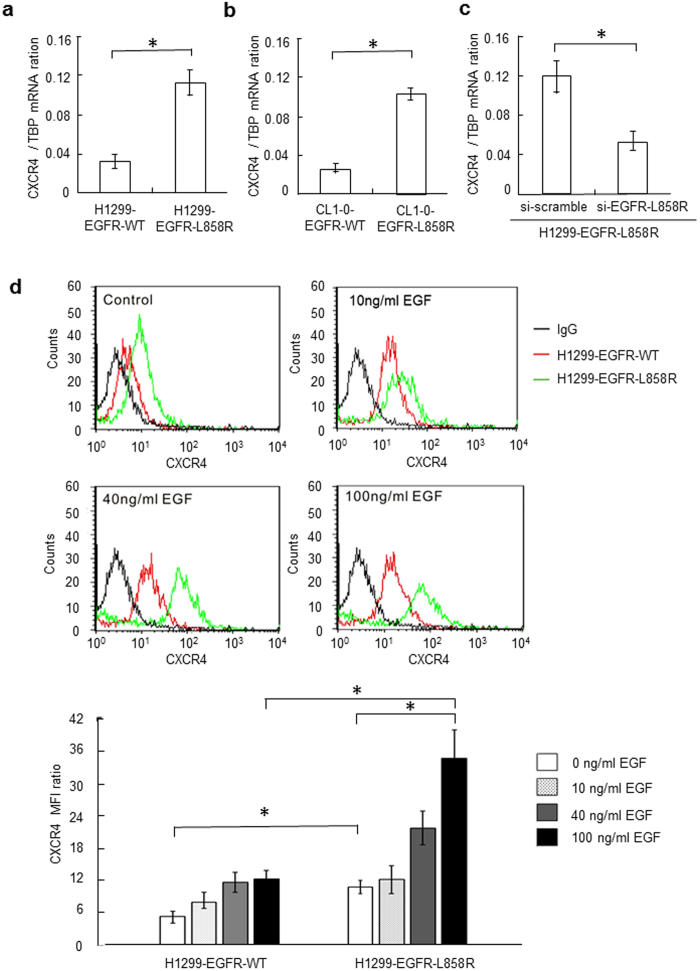
The EGFR-L858R mutant increases expression of the chemokine receptor, CXCR4. (**a**) CXCR4 mRNA expression in H1299-EGFR-WT and H1299-EGFR-L858R cells was quantified by real-time RT-PCR (*P < 0.01). (**b**) CXCR4 mRNA expression in CL1-0-EGFR-WT and CL1-0-EGFR-L858R cells was quantified by real-time RT-PCR (*P < 0.01). (**c**) CXCR4 mRNA expression in H1299-EGFR-L858R cells after transfection with EGFR-L858R–specific siRNA or scrambled siRNA control was quantified by real-time RT-PCR (*P < 0.01). (**d**) H1299-EGFR-WT and H1299-EGFR-L858R cells were serum-starved for 24 hours, and then treated with different concentrations of EGF (10, 40 and 100 ng/ml) for 2 hours. After EGF stimulation, CXCR4 surface expression was evaluated by flow cytometry. Data are presented as means ± SD of three independent experiments. *P*-values (two-sided) were determined using Student’s t-test.

Quantitative real-time reverse transcription-polymerase chain reaction (RT-PCR) and flow cytometric analysis were performed to validate the elevated expression of CXCR4 obtained from GEO public database (GSE11729). As shown in Fig. 3a, the basal expression level of CXCR4 increased at least 3-fold in H1299-EGFR-L858R cells compared to control H1299-EGFR-WT cells (*P* < 0.01). The expression level of CXCR4 increased at least 3.4-fold in CL1-0-EGFR-L858R cells compared to control CL1-0-EGFR-WT cells (P < 0.01, Fig. 3b). We also found that siRNA-mediated knockdown of EGFR-L858R in H1299-EGFR-L858R cells significantly decreased CXCR4 mRNA levels (*P* < 0.01, Fig. 3c). To investigate whether activation of EGFR signaling pathways regulated CXCR4 expression, we stimulated H1299-EGFR-WT and H1299-EGFR-L858R cells with different concentrations of EGF (10, 40 and 100 ng/ml) and examined changes in CXCR4 surface expression using flow cytometry. Stimulation with EGF for 24 hours induced a concentration-dependent increase in cell surface expression of CXCR4 protein in both cell lines. At a concentration of 100 ng/ml, EGF increased CXCR4 surface levels in H1299-EGFR-WT and H1299-EGFR-L858R by 2.1- and 3.4-fold, respectively (Fig. 3d), indicating a strong positive relationship between activation of the EGFR signaling pathway and expression of CXCR4.

### EGFR-L858R–dependent increases in CXCR4 expression are mediated by the ERK pathway

**Figure 4:**
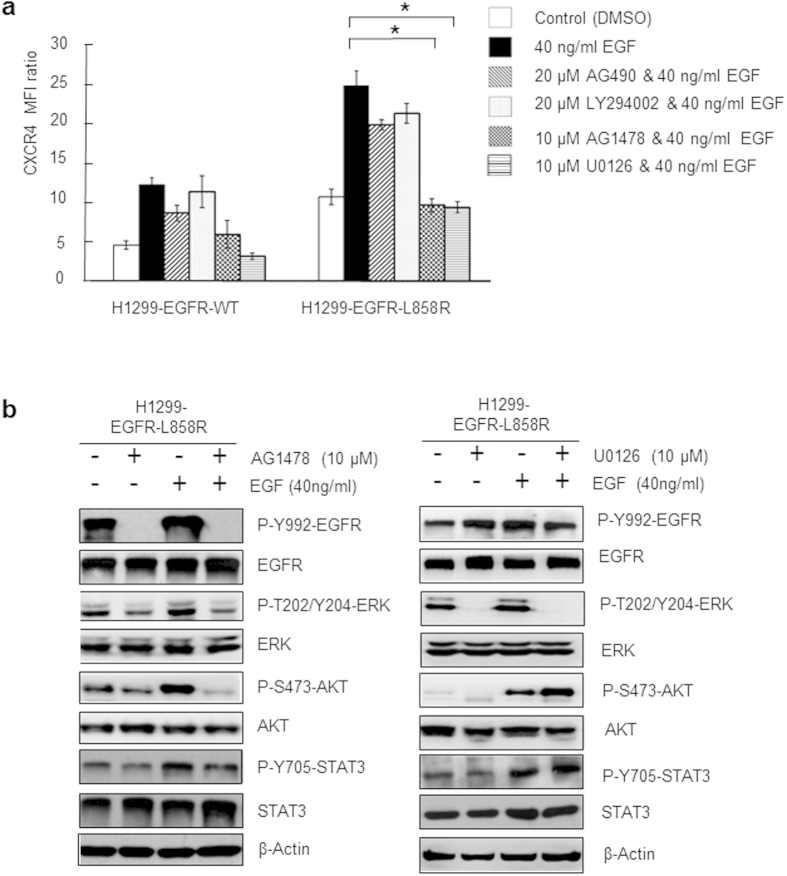
EGFR-L858R–dependent up-regulation of CXCR4 expression in lung cancer cells is mediated by the ERK signaling pathway. H1299-EGFR-L858R cells were serum-starved for 24 hours, preincubated for 1 hour with or without pharmacological inhibitors of individual pathways, and then stimulated with EGF (40 ng/ml) for 5 minutes. (a) Surface CXCR4 protein expression was evaluated by flow cytometry. CXCR4 expression data are presented as MFI ratio, and are expressed as means ± SD of three independent experiments (*P < 0.01). *P*-values (two-sided) were determined using Student’s t-test. (**b**) The effective inhibition concentration of EGFR inhibitor AG1478 (10 μM) were used and the phosphorylated and total protein of EGFR, ERK, AKT, and STAT3 were detected by Western blotting in H1299-EGFR-L858R cells with or without EGF stimulation. The full size blots were shown in the Supplementary Figure S4 and band of interest is indicated with an arrow. The samples derive from the same experiment and that blots were processed in parallel. (**c**) The effective inhibition concentration of ERK inhibitor U0126 (10 μM) were used and the phosphorylated and total protein of EGFR, ERK, AKT, and STAT3 were detected by Western blotting in H1299-EGFR-L858R cells with or without EGF stimulation. The full size blots were shown in the Supplementary Figure S4 and band of interest is indicated with an arrow. The samples derive from the same experiment and that blots were processed in parallel.

To identify downstream signaling pathways involved in EGFR-L858R–dependent expression of CXCR4, the effective inhibition concentration of pharmacological inhibitors (10 μM EGFR inhibitor AG1478, 10 μM ERK inhibitor U0126, 20 μM PI3K-AKT inhibitor LY294002 and 20 μM JAK-STAT3 inhibitor AG490) were performed in H1299-EGFR-L858R cells and then stimulated with 40 ng/ml EGF. As showed in Supplementary Figure S3, the effective inhibition concentration of each inhibitors abolished phosphorylation (activation) of their target protein in H1299-EGFR-L858R cells with 40 ng/ml EGF-stimulated. AG1478 and U0126 exerting marked inhibitory effects at 10 μM, and LY294002 and AG490 exerting similar inhibitory effects at 20 μM. As shown in Fig. 4a, EGFR-L858R–dependent increases in CXCR4 expression were markedly abrogated by 10 μM EGFR inhibitor AG1478 and 10 μM ERK inhibitor U0126. In contrast, the 20 μM PI3K-AKT inhibitor LY294002 and the 20 μM JAK-STAT3 inhibitor AG490 can effectively inhibit the phosphorylated-AKT and phosphorylated-STAT3, but exerted only minimal inhibitory effects (<20%) on the EGFR-L858R–dependent CXCR4 expression. The results indicated that PI3K-AKT and JAK-STAT3 pathways are not required for EGFR-L858R–dependent CXCR4 expression. To affirm the role of ERK pathway involved in EGFR-L858R–dependent CXCR4 expression, the effective inhibition concentration of EGFR inhibitor AG1478 and ERK inhibitor U0126 were used and the phosphorylated and total protein of EGFR, ERK, AKT, and STAT3 were detected by Western blotting in H1299-EGFR-L858R cells. The results showed that EGFR inhibitor AG1478 were significantly inhibition the phosphorylated-EGFR, phosphorylated-ERK, phosphorylated-AKT, but not phosphorylated-STAT3 (Fig. 4b). Using ERK inhibitor U0126 treated the H1299-EGFR-L858R cells, indicated that only phosphorylated-ERK was abolished (Fig. 4c). These findings suggest that the EGFR-L858R mutant promotes the expression of CXCR4 in lung cancer cells primarily through EGFR and ERK signaling pathways.

### The CXCL12-CXCR4 axis is required for EGFR-L858R–dependent invasive properties

**Figure 5:**
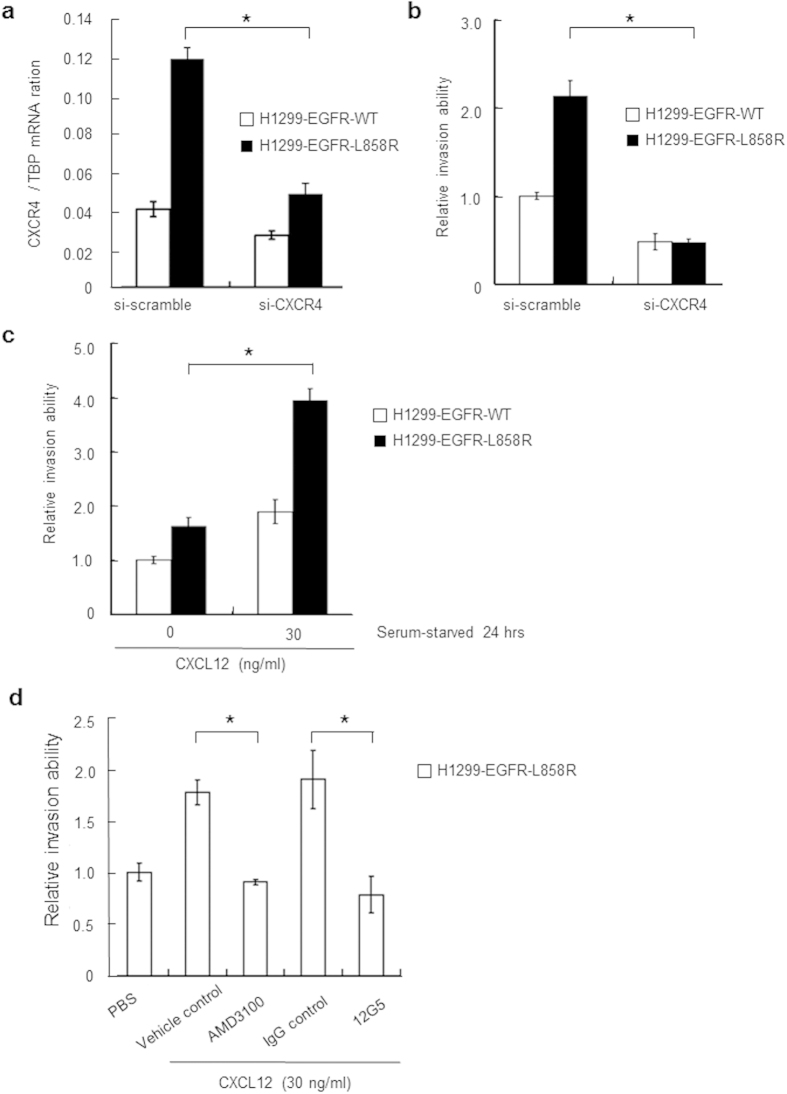
The CXCL12-CXCR4 axis is required for EGFR-L858R–dependent increases in invasive properties. (**a**) CXCR4 in H1299-EGFR-L858R cells was knocked down using a specific siRNA. Cells were harvested 48 hours after transfection, and CXCR4 expression was analyzed by real-time RT-PCR (*P < 0.01). si-Scramble, cells transfected with scrambled control siRNA; si-CXCR4, cells transfected with CXCR4-specific siRNA. (**b**) The invasion ability of H1299-EGFR-L858R cells was determined using a modified Boyden chamber after siRNA-mediated knockdown of CXCR4 (*P < 0.01). (**c**) The EGFR-L858R mutant enhances CXCL12-CXCR4–mediated cell invasiveness. H1299-EGFR-WT and H1299-EGFR-L858R cells were serum-starved for 24 hours, after which invasion ability was assessed in the absence or presence of CXCL12 (30 ng/ml) (*P < 0.01). (**d**) H1299-EGFR-L858R cells were pretreated with a CXCR4 neutralizing antibody (12G5), isotype control antibody (IgG control), or CXCR4 antagonist (AMD3100) for 30 minutes. The invasion ability of cancer cells in the presence of CXCL12 (30 ng/ml) was then evaluated using a modified Boyden chamber assay. The results are presented as changes relative to the control expressed as means ± SD of three independent experiments. *P*-values (two-sided) were determined using Student’s t-test.

To determine if CXCR4 is indeed the major determinant of the EGFR-L858R–dependent increase in invasion potential, we used a CXCR4 siRNA approach. Specific siRNAs targeting CXCR4 effectively knocked down CXCR4 transcript levels and suppressed the invasion ability of H1299-EGFR-WT and H1299-EGFR-L858R cells (Fig. 5a,b). The cell proliferation rate were also detected using MTT assay. CXCR4 in H1299-EGFR-WT and H1299-EGFR-L858R cells was knocked down using a CXCR4 specific siRNA. Cells were harvested 24 hours after transfection, and then the cell proliferation rates of H1299-EGFR-WT-siScramble, H1299-EGFR-WT-siCXCR4, H1299-EGFR-L858R-siScramble and H1299-EGFR-L858R-siCXCR4 lung cancer cells were analyzed. The proliferation rates of these four cells have no significant difference (Supplementary Figure S1). We assayed invasion responses of H1299-EGFR-WT and H1299-EGFR-L858R cells to stimulation with CXCL12 (30 ng/ml) in serum-free medium. These experiments showed that CXCL12-induced invasion ability was greater in cells harboring the EGFR-L858R mutation than in EGFR-WT control cells (Fig. 5c). We further examined the role of the CXCL12-CXCR4 axis in the EGFR-L858R–dependent increase in invasion ability by pretreating H1299-EGFR-L858R cells with a CXCR4 neutralizing antibody (12G5) or CXCR4 antagonist (AMD3100) and then stimulating with CXCL12. Blocking CXCR4 activation through either approach significantly suppressed the effect of CXCL12 on H1299-EGFR-L858R cell invasion (P < 0.01, Fig. 5d). Taken together, these results indicate that the CXCL12-CXCR4 pathway plays an important role in the EGFR-L858–dependent increase in invasion ability.

### CXCR4 expression in lung adenocarcinoma patients harboring the EGFR-L858R mutation

**Figure 6:**
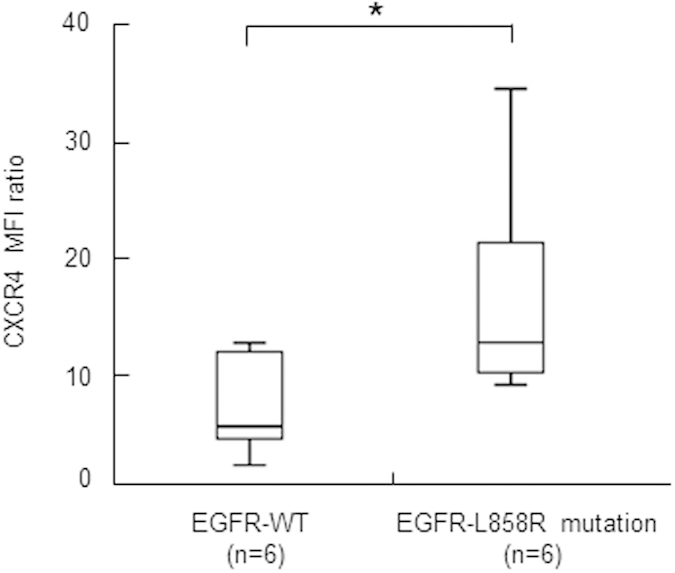
CXCR4 expression in lung adenocarcinoma patients according to EGFR-L858R mutation status. The CXCR4 surface protein expression in lung adenocarcinoma cells from EGFR-L858R and EGFR-WT tumors was determined by flow cytometry. Box shows median and interquartile range ± 95% confidence interval. Patient samples were tested in triplicate (*P = 0.025, Mann-Whitney U Test).

To confirm the association between CXCR4 and the EGFR-L858R mutation, we performed an EGFR mutation analysis of cancer cells in MPEs from 12 lung adenocarcinomas and analyzed the surface expression of CXCR4 by flow cytometry. This analysis included six patients with EGFR-WT and six patients with the EGFR-L858R mutation. As shown in Fig. 6A, surface expression of CXCR4 protein on lung cancer cells harboring the EGFR-L858R mutation (median, 12.61; range: 8.95–34.56) was significantly higher than that in EGFR-WT cells (median, 5.36; range, 2.00–12.58; *P* = 0.025, Mann-Whitney U Test). The clinical characteristics of the 12 patients, shown in supplementary Table S1, were not different between patients with EGFR-WT and those with EGFR-L858R.

## Discussion

In this study, we show that the EGFR-L858R mutant increases lung adenocarcinoma cell invasion ability and promotes formation of MPE. Specifically, we found that ectopic expression of EGFR-L858R in lung cancer cells acted through activation of ERK signaling pathways to induce the expression of CXCR4. We further found that this increase in CXCR4 expression was central to the effects of EGFR-L858R, showing that siRNA-mediated knockdown of CXCR4 or inhibition of CXCR4 with a neutralizing antibody or antagonist significantly suppressed the invasion ability of H1299-EGFR-L858R cells. Finally, we demonstrated that clinical samples of lung adenocarcinomas harboring the EGFR-L858R mutation exhibited increased surface expression of CXCR4. Collectively, our findings indicate that the EGFR-L858R mutant increases cancer cell invasive ability and MPE formation through ERK-dependent activation of the CXCL12-CXCR4 axis.

Expression of EGFR is significantly elevated in a variety of cancer types^36^, and activation of EGFR signaling pathways has been implicated in the development and progression of cancer^37^,^38^. It has been shown that EGFR mutational analyses of MPE are feasible and EGFR mutation status correlates with response to targeted therapy with TKIs^14^,^15^,^16^. Previous reports have also shown that the frequency of EGFR mutations is increased in Taiwan and Dutch cohorts of lung adenocarcinoma patients with associated MPE^15^,^17^. These results suggest that lung adenocarcinoma cells harboring the EGFR-L858R mutation tend to invade the adjacent pleural cavity and are involved in MPE formation. However, no studies have directly addressed the role of the EGFR-L858R mutation in lung cancer cell invasion and MPE formation. Here, using gain- and loss-of-function approaches, we demonstrated that the EGFR-L858R mutant enhances cancer cell invasion compared with EGFR-WT. We showed that the invasive activity of H1299-EGFR-L858R cells was significantly greater than that of control H1299-EGFR-WT cells, and was decreased by knocking down EGFR-L858R expression (Fig. 1). We also found that lung cancer cells harboring the EGFR-L858R mutation promoted the formation of MPE *in vivo* (Fig. 2). Thus, this study is the first to demonstrate that the EGFR-L858R mutant enhances the formation of MPE associated with lung cancer.

Chemokines are frequently involved in cellular interactions and tropism under inflammation-associated conditions^39^. Notably, recent studies have highlighted the importance of chemokines and chemokine receptors in inflammation associated with carcinogenesis. Chemokine receptors and their corresponding ligands have been demonstrated to make a significant contribution to cancer cell survival, growth, migration, invasion, and angiogenesis^40^,^41^. To date, at least 20 human chemokine receptors have been identified—all members of the G protein-coupled receptor superfamily; these are subdivided into CXCR (7 members), CCR (11 members), CR (1 member), CX3CR (1 member), and other subfamilies^41^. It is clear from large clinical studies that selected chemokine receptors are often up-regulated in a variety of common human cancers, including lung cancer, breast cancer, ovarian cancer, colorectal cancer, and malignant melanoma^40^. Accumulating evidence suggests that, in addition to being involved in cancer development and metastasis, chemokine receptors are important regulators of the pathogenesis of MPE^23^,^26^. As part of our investigation of the possible molecular mechanisms involved in the formation of MPE associated with EGFR-L858R–mutant lung adenocarcinoma, we used public GEO data (GSE11729), real-time RT-PCR, and flow cytometric analyses to determine whether chemokine receptors are differentially expressed between H1299-EGFR-L858R and H1299-EGFR-WT cells. We found that basal expression levels of the chemokine receptor, CXCR4, were elevated in H1299-EGFR-L858R cells compared to control H1299-EGFR-WT cells. We also indicated that EGF stimulated a significant increase in the expression of CXCR4 in lung adenocarcinoma cells harboring the EGFR-L858R mutation (Fig. 3).

Activation of the EGFR has been reported to promote cell survival, proliferation, invasion, and metastasis through activation of JAK-STAT, PI3K-AKT, and MAPK (mitogen-activated protein kinase) pathways^37^,^38^. To better understand how CXCR4 expression is induced in H1299-EGFR-L858R cells, we analyzed the phosphorylation status of the main downstream effectors of EGF signaling pathways—EGFR, AKT, ERK1/2 and STAT3—using pharmacological inhibitors in conjunction with Western blotting. Our data suggest that the EGFR-L858R mutant promotes the expression of CXCR4 in lung cancer cells primarily through the ERK signaling pathway, rather than PI3K-AKT and JAK-STAT3 pathways (Fig. 4), thereby increasing invasive ability.

Over the past decade, accumulating evidence has demonstrated that the chemokine, CXCL12, and it cognate receptor, CXCR4, contribute to metastasis and clinical outcomes in many different types of solid cancers, including lung cancer^42^. It has also been shown that a number of NSCLC cell lines express high levels of CXCR4, which is associated with aggressive behavior, and that CXCL12-activated CXCR4 promotes migration and invasion of these cell lines *in vitro*^43^. Furthermore, preferred lung cancer metastasis sites *in vivo* exhibit significantly higher levels of CXCL12 protein compared with those observed at the primary tumor site or in plasma, indicating that a chemotactic gradient may be established between the site of the primary tumor and metastatic sites^43^. The attraction between CXCL12 and CXCR4 causes breast cancer cells to leave the circulation and migrate into organs containing large amounts of chemokines, where the cancer cells proliferate, induce angiogenesis, and form metastatic tumors^44^,^45^. CXCR4 is also involved in the metastasis of prostate cancer to the bone marrow^46^ and of colon cancer to the liver^47^. In the current study, we reported that CXCR4 surface protein expression in clinical samples from lung cancer patients with tumors expressing the EGFR-L858R mutation is significantly higher than that in tumors expressing EGFR-WT (Fig. 6). We extended these findings using CXCR4-specific siRNA, neutralizing antibody, and antagonist (AMD3100) to demonstrate a role for the CXCL12-CXCR4 axis in mediating the EGFR-L858-dependent increase in invasion ability.

In summary, our findings demonstrate that lung adenocarcinomas harboring the EGFR-L858R mutation exhibit increased cancer cell invasive ability and enhanced MPE formation, effects mediated through ERK-dependent activation of CXCL12-CXCR4 signaling pathways. Our results further suggest that targeting the production of CXCR4 and blocking the CXCL12-CXCR4 pathway might be effective strategies for treating NSCLCs harboring specific types of EGFR mutations.

## Methods

### Cell culture and plasmid

The human lung carcinoma cell lines, H1299-EGFR-WT (overexpressing EGFR-WT) and H1299-EGFR-L858R (overexpressing EGFR-L858R), and the expression vectors pcDNA4-EGFR-WT (EGFR-WT in pcDNA4 expression vector) and pcDNA4-EGFR-L858R (EGFR-L858R in pcDNA4 expression vector) kindly provided by Dr. Yi-Rong Chen (Division of Molecular and Genomic Medicine, National Health Research Institutes, Taiwan)^27^.

H1299-EGFR-WT and H1299-EGFR-L858R were cultured in RPMI-1640 medium (Sigma-Aldrich, Louis, MO) supplemented with 10% fetal bovine serum (FBS), 100 units/ml penicillin, 100 ng/ml streptomycin, and 500 μg/ml of zeocin (Invitrogen, Carlsbad, CA). The low invasive ability of human lung adenocarcinoma cell lines (CL1-0 cells) were isolated from a 64-year-old male patient with a poorly differentiated adenocarcinoma and selected in our laboratory as previously described^48^.

### Transfection of siRNA and real-time RT-PCR

Transfections were performed using Lipofectamine 2000 reagent (Invitrogen) according to the manufacturer’s instructions. The CXCR4 siRNA duplexes were purchased from Ambion (Austin, TX). Total mRNA was extracted using TRIzol reagent (Invitrogen). cDNA was synthesized from total RNA by reverse transcription using Superscript III reverse transcriptase (Invitrogen), as described by the manufacturer. CXCR4 primers and probes were purchased from Applied Biosystems (Foster City, CA). EGFR-WT and EGFR-L858R were quantified by real-time RT-PCR (TaqMan) using the primers 5′-CCGCAGCATGTCAAGATCAC-3′ (forward) and 5′-TCCTTCTGCATGGTATTCTTTCTCT-3′ (reverse)^49^ and the TaqMan probes 5′-VIC-TTGGCCAGCCCAA-MGB-3′ (EGFR-WT) and 5′-FAM-TTGGCCCGCCCAA-MGB-3′ (EGFR-L858R). Reactions were performed on an Applied Biosystems 7500 Real-Time PCR System using TaqMan EZ RT-PCR Core Reagents (Applied Biosystems).

### Antibodies and Western blotting

After serum-starving for 24 hours, H1299-EGFR-WT and H1299-EGFR-L858R cells were treated with 40 ng/ml EGF for 5 minutes, with or without pretreatment with the pharmacological inhibitors, AG1478 (EGFR inhibitor), AG490 (JAK-STAT3 inhibitor), LY294002 (PI3K-AKT inhibitor), U0126 (ERK inhibitor) or vehicle control (DMSO) for 1 hour. Cells lysates were prepared and analyzed by Western blotting as described previously^50^. The following primary antibodies were used for Western blot analyses: anti-EGFR (Santa Cruz Biotechnology, Santa Cruz, CA), anti-phospho-Y992-EGFR (Cell Signaling Technology, Beverly, MA), anti-phospho-Y705-STAT3 (Cell Signaling Technology), anti-STAT3 (Cell Signaling Technology), anti-phospho-S473-AKT (Cell Signaling Technology), anti-AKT (Cell Signaling Technology), anti-ERK1/2 (Invitrogen), and anti-phospho-T202/Y204-ERK (BD Biosciences, San Diego, CA). Recombinant human EGF, CXCL12 (SDF-1α), CXCR4 neutralizing antibody (12G5), monoclonal anti-CXCR4 antibody, and mouse IgG2B isotype control antibody were purchased from R&D Systems (Minneapolis, NM). Chemiluminescent signals on western blots were captured using the Fujifilm LAS 3000 system (Fujifilm) and image processing was performed with Adobe Photoshop software (Adobe Systems).

### Invasion assay

Invasion assays were performed as previously described using 8-μm-pore-size Costar Transwell chambers (Corning, NY) and Transwell filters coated with a thin layer of BD Matrigel (BD Biosciences)^50^. Prior to invasion assay, cells were pretreated for 30 minutes with inhibitors, including the CXCR4 neutralizing antibody (12G5), isotype control antibody, or AMD3100 (Sigma-Aldrich). Cells (2 × 10^4^) were suspended in 200 μl of serum-free RPMI-1640 medium placed in the upper chamber; serum-free RPMI-1640 medium containing 30 ng/ml CXCL12 was placed in the lower chamber. Cells were incubated for 18 hours and then removed from the upper surface of the filter by scraping with a cotton swab. The cells that adhered to the bottom of the membrane were stained with Giemsa solution (Sigma-Aldrich).

### Flow cytometry

Flow cytometric analyses of surface CXCR4 expression on cultured cancer cells were performed according to the manufacturer’s protocol (R&D Systems). In brief, adherent cells, detached from culture dishes by exposure to 0.5 mM EDTA, were pre-incubated with human IgG (1 μg per 10^6^ cells) for 15 minutes at room temperature. Cells were then stained with phycoerythrin-conjugated mouse monoclonal anti-human CXCR4 antibody or an isotype-matched control antibody (R&D Systems). After incubation, cells were washed with phosphate-buffered saline (PBS) containing 0.05% bovine serum albumen (BSA) and resuspended in ~300–400 μl of PBS. Data were collected using a FACSCalibur flow cytometer (BD Biosciences) and analyzed using CellQuest software (BD Biosciences). CXCR4 expression data were presented as fold increase in mean fluorescence intensity (MFI) relative to isotype control staining (MFI ratio).

### Animal model and malignant pleural effusion formation

All animal works were performed under protocols approved by the Institutional Animal Care and Use Committee of the College of Medicine, National Taiwan University. The methods were carried out in accordance with the approved guidelines. Four-to-six-week-old NOD/SCID male mice were obtained from the Animal Center of National Taiwan University.

Intrapleural injections were performed as described previously^29^,^30^. The tumor cells were injected into the left pleural cavity of the mice using a syringe with a 27 gauge needle. The mice were randomly divided into two groups to receive injections of H1299-EGFR-WT or H1299-EGFR-L858R cells. Mice (n = 5/group) were injected with 1.5 × 10^5^ tumor cells in 50 μl PBS, and sacrificed by CO_2_ asphyxiation after 14 days.

### Patients and sample collection

Human samples were collected and archived under protocols approved by Institutional review board (IRB) of National Taiwan University Hospital. All written informed consent documents regarding the use of these specimens for future molecular studies were completed before thoracentesis. The methods were carried out in accordance with the approved guidelines. Pleural effusions were collected from patients who received thoracentesis in the chest ultrasonography examination room of National Taiwan University Hospital from May to July, 2009. Malignant pleural effusions were confirmed by cytology examination. Some of the samples were previously examined and reported in studies of EGFR mutations. For flow cytometry analyses of CXCR4 surface expression levels, only lung adenocarcinoma patients with EGFR-L858R or EGFR-WT were enrolled. MPE was managed as described previously^50^.

### Statistical analysis

Student’s t-test was used to compare the means of two groups. Two-sided P-values less than 0.05 were considered significant. All analyses were performed using SPSS software version 15.0 for Windows (SPSS Inc., Chicago, IL).

## Additional Information

**How to cite this article**: Tsai, M.-F. *et al*. EGFR-L858R mutant enhances lung adenocarcinoma cell invasive ability and promotes malignant pleural effusion formation through activation of the CXCL12-CXCR4 pathway. *Sci. Rep*. **5**, 13574; doi: 10.1038/srep13574 (2015).

## Supplementary Material

Supplementary Information

## Figures and Tables

**Table 1:** Fold changes in chemokine receptor expression in H1299-EGFR-L858R versus H1299-EGFR-WT cells.

Chemokine receptor	Fold Change	
	without EGF stimulation	EGF stimulation
CXCR1	<1.5	<1.5
CXCR2	<1.5	<1.5
CXCR3	<1.5	<1.5
CXCR4	1.6	2.6
CXCR5	1.8	<1.5
CXCR6	2.0	<1.5
CXCR7	<1.5	<1.5
CCR1	<1.5	<1.5
CCR2	<1.5	<1.5
CCR3	<1.5	<1.5
CCR4	<1.5	<1.5
CCR5	<1.5	<1.5
CCR6	<1.5	<1.5
CCR7	<1.5	<1.5
CCR8	<1.5	<1.5
CCR9	<1.5	<1.5
CCR10	<1.5	<1.5
XCR1	<1.5	<1.5
CX3CR1	<1.5	2.3
