# Supplementary material for: EGFR-L858R mutant enhances lung adenocarcinoma cell invasive ability and promotes malignant pleural effusion formation through activation of the CXCL12-CXCR4 pathway
Source: Sci Rep. 2015 Sep 4;5:13574. doi: 10.1038/srep13574 (PMC4559673; doi:10.1038/srep13574)
Supplement: Supplementary Information [file srep13574-s1.pdf]

Supplementary Information

EGFR-L858R mutant enhances lung adenocarcinoma cell invasive ability and promotes malignant pleural effusion formation through activation of the CXCL12-CXCR4 pathway

Meng-Feng Tsai [1*], Tzu-Hua Chang [2*], Shang-Gin Wu [3], Hsiao-Yin Yang [2], Yi-Chiung Hsu[5], Pan-Chyr Yang [2,4] & Jin-Yuan Shih [2,4]

Affiliation of authors:
1Department of Molecular Biotechnology, College of Biotechnology and Bioresources, Dayeh University, Changhua 51591, Taiwan,
2Department of Internal Medicine, National Taiwan University Hospital, and College of Medicine, National Taiwan University, Taipei 10002, Taiwan,
3Department of Internal Medicine, National Taiwan University Hospital, Yun-Lin Branch, Yunlin 64041, Taiwan,
4 Graduate Institute of Clinical Medicine, College of Medicine, National Taiwan University, Taipei 10002, Taiwan,
5Institute of Statistical Science, Academia Sinica, Taipei 11529, Taiwan.

*These two authors contributed equally to this work.
Meng-Feng Tsai & Tzu-Hua Chang

Corresponding author:
Jin-Yuan Shih, MD, PhD,
Department of Internal Medicine, National Taiwan University Hospital, College of Medicine, National Taiwan University, 7 Chung-Shan South Road, Taipei 10002, Taiwan.
Telephone: +886-2-23123456 ext. 62905
Fax: +886-2-2358-2867
E-mail address: jyshih@ntu.edu.tw

Supplementary Figure S1
Supplementary Figure S2
Supplementary Figure S3
Supplementary Figure S4
Supplementary Table S1

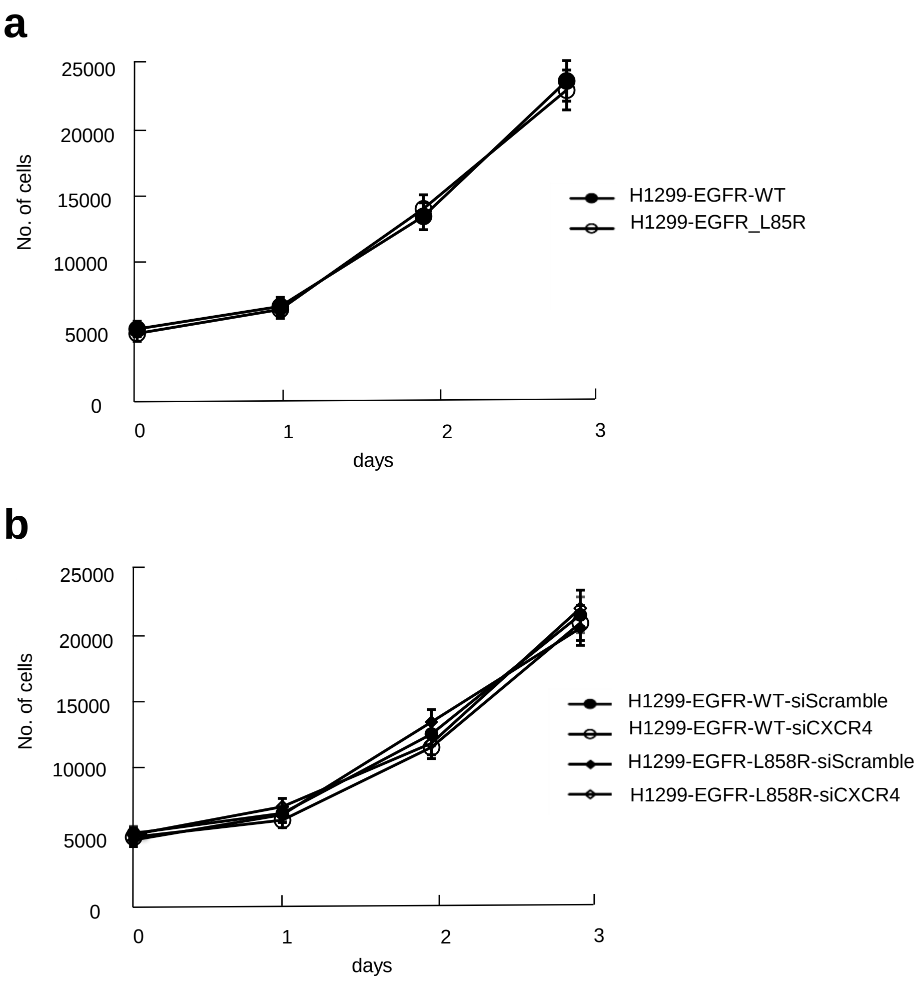

Figure S1. Cancer cell proliferation rates assessed by thiazolyl blue tetrazolium bromide (MTT) assay. (a) The cell proliferation rates of H1299-EGFR-WT and H1299- EGFR-L858R lung cancer cells. The proliferation rates of these two cells have no difference. (b) CXCR4 in H1299-EGFR-WT and H1299-EGFR-L858R cells was knocked down using a CXCR4 specific siRNA. Cells were harvested 24 hours after transfection, and than the cell proliferation rates of H1299-EGFR-WT-siScramble, H1299-EGFR-WT-siCXCR4, H1299-EGFR-L858R-siScramble and H1299- EGFR-L858R-siCXCR4 lung cancer cell were analyzed. siScramble, cells transfected with scrambled control siRNA; siCXCR4, cells transfected with CXCR4-specific siRNA. The proliferation rates of these four cells have no difference.

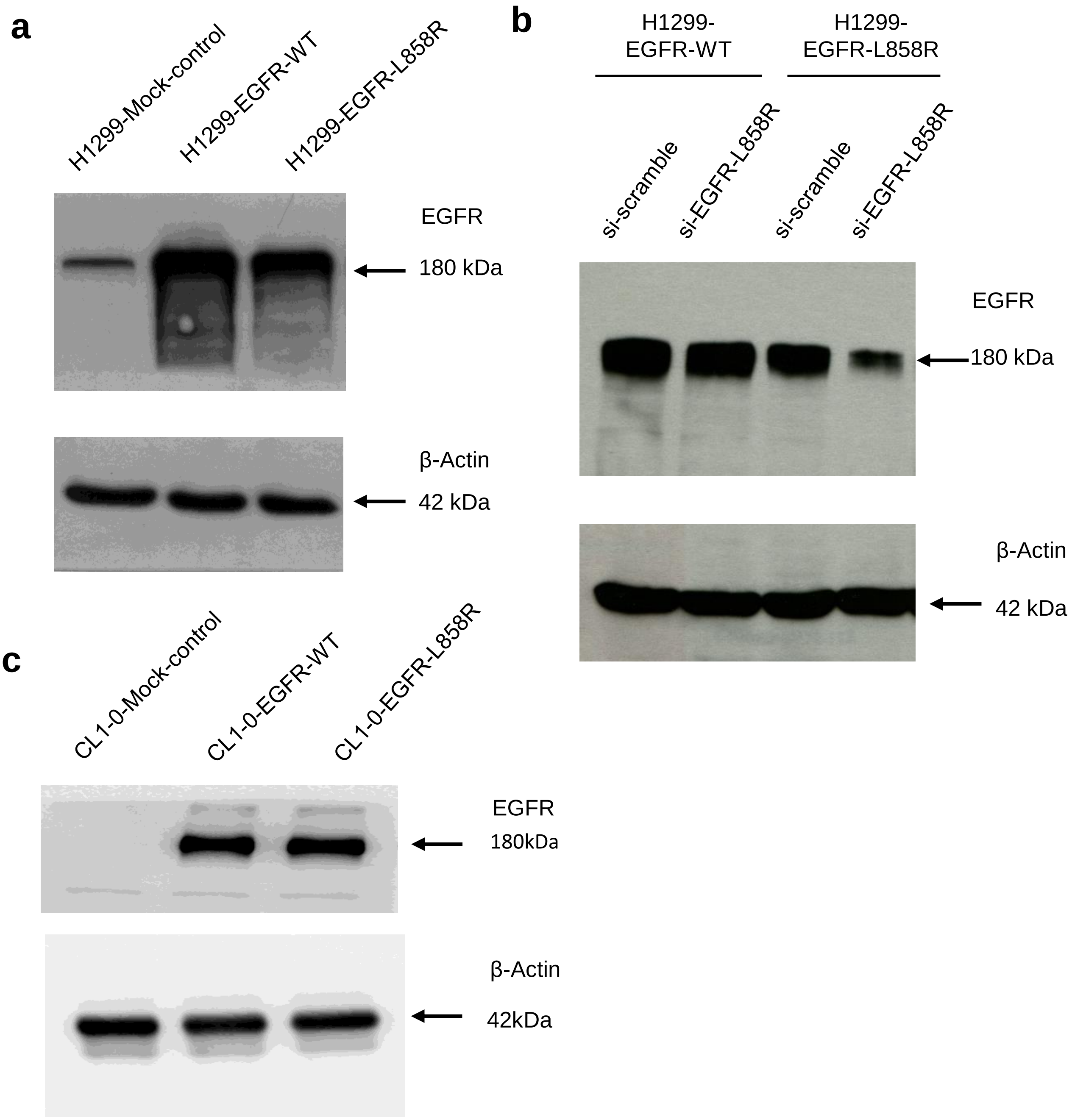

Figure S2 EGFR-L858R expression in lung cancer cell. (a) Over-expression of EGFR-WT and EGFR-L858R in lung adenocarcinoma H1299 cells was evaluated by Western blotting. (b) Small interfering RNA (siRNA) specifically targeting EGFR-L858R knocked down EGFR-L858R protein expression in H1299-EGFR-L858R cells. (c) Over-expression of EGFR-WT and EGFR-L858R in lung adenocarcinoma CL1-0 cells was evaluated by Western blotting. This is a full length image of the cropped blot presented in the Figure 1a, c and e.

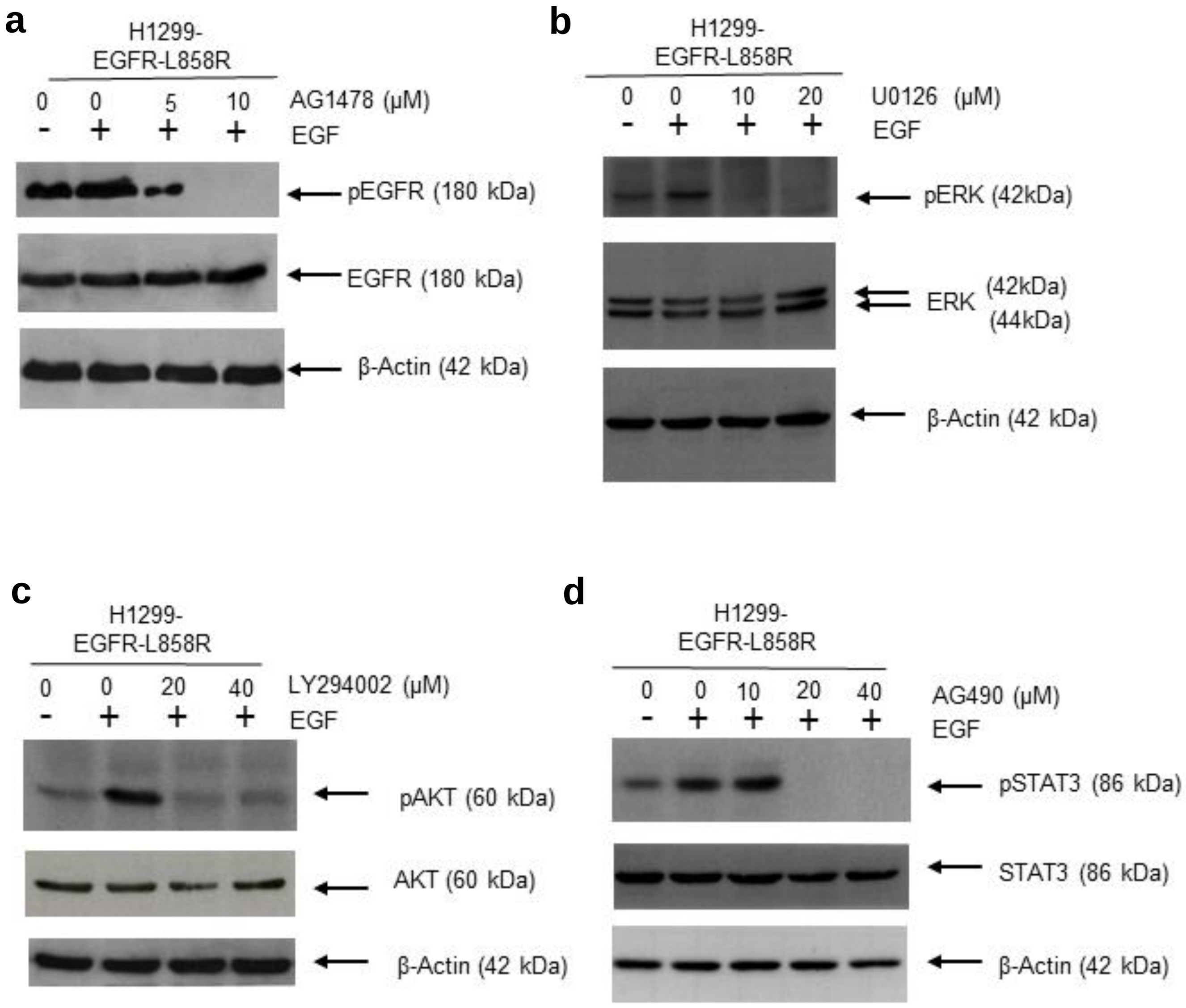

Figure S3 The effective inhibition concentration of pharmacological inhibitors (EGFR inhibitor AG1478, ERK inhibitor U0126, PI3K-AKT inhibitor LY294002 and JAK-STAT3 inhibitor AG490) were performed in H1299-EGFR-L858R cells and then stimulated with 40ng/ml EGF. The phosphorylation status of EGFR, AKT, ERK1/2 and STAT3 (the main downstream effectors of EGF signaling pathways) was determined by Western blotting. (a) EGFR and phospho-EGFR levels were evaluated in H1299-EGFR-L858R cells pretreated with different concentrations of the EGFR inhibitor, AG1478. (b) ERK and phospho-ERK levels were evaluated in H1299-EGFR-L858R cells pretreated with different concentrations of the ERK inhibitor, U0126. (c) AKT and phospho-AKT levels were evaluated in H1299-EGFR-L858R cells pretreated with different concentrations of the PI3K-AKT inhibitor, LY294002. (d) STAT3 and phospho-STAT3 levels were evaluated in H1299-EGFR-L858R cells pretreated with different concentrations of the JAK-STAT3 inhibitor, AG490.

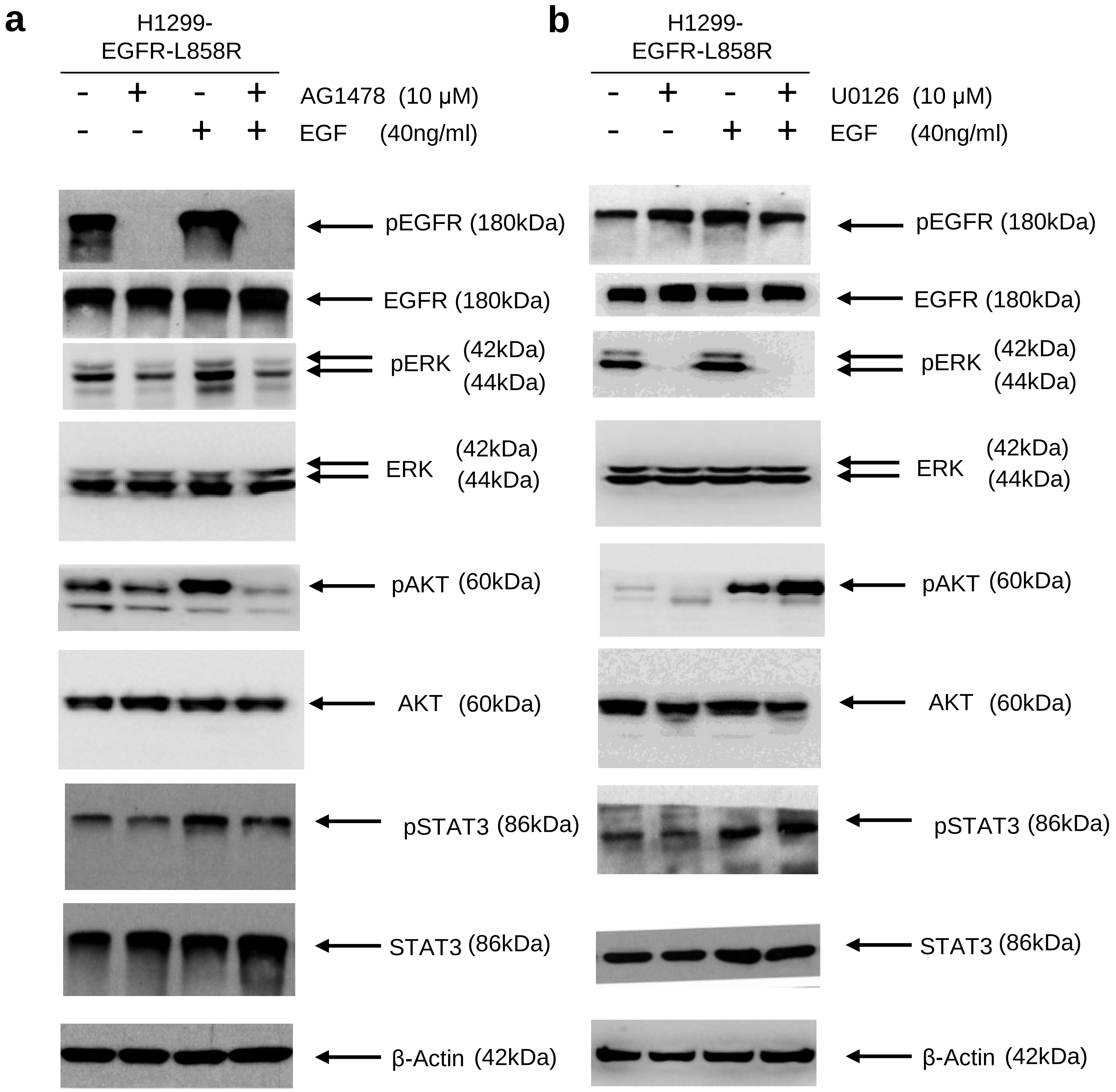

Figure S4. (a) The effective inhibition concentration of EGFR inhibitor AG1478 (10um) were used and the phosphorylated and total protein of EGFR, ERK, AKT, and STAT3 were detected by Western blotting in H1299-EGFR-L858R cells with or without EFG stimulation. (b) The effective inhibition concentration of ERK inhibitor U0126 (10uM) were used and the phosphorylated and total protein of EGFR, ERK, AKT, and STAT3 were detected by Western blotting in H1299-EGFR-L858R cells with or without EFG stimulation.The results showed that EGFR inhibitor AG1478 were significantly inhibition the phosphorylated-EGFR, phosphorylated-ERK, phosphorylated-AKT, but not phosphorylated-STAT3. Using ERK inhibitor U0126 treated the H1299-EGFR-L858R cells, indicated that only phosphorylated-ERK was abolished This is a full length image of the cropped blot presented in the Figure 4b,c.

Table S1: Clinical characteristics of the 12 lung adenocarcinoma patients with malignant pleural effusion (MPE)

Variable		L848R	WT	P-value
Total No.		6	6	
Sex				
	Male	4	4	1.000
	Female	2	2	
Age				
	Median	68.2	70.0	0.631*
	(Range)	(55.7–79.8)	(60.1–77.4)	
Smoking				
	Never	3	3	1.000
	Current/Former	3	3	
T				0.164
	1	0	2	
	2	1	2	
	4	5	2	
N				0.494
	0	0	1	
	1	1	0	
	2	2	3	
	3	3	2	

*By Mann-Whitney U test

To confirm the association between CXCR4 and the EGFR-L858R mutation, we performed an EGFR mutation analysis of cancer cells in MPEs from 12 lung adenocarcinomas and analyzed the surface expression of CXCR4. The clinical characteristics of the 12 patients, shown in supplementary Table S1.
